# The therapeutic effect of adipose-derived lipoaspirate cells in femoral head necrosis by improving angiogenesis

**DOI:** 10.3389/fcell.2022.1014789

**Published:** 2022-10-18

**Authors:** Weixin Zhang, Cheng Zheng, Tiefeng Yu, Houjian Zhang, Jiaxin Huang, Liyue Chen, Peijian Tong, Gehua Zhen

**Affiliations:** ^1^ Department of Traditional Chinese Medical Orthopedic Surgery, Zhejiang Chinese Medical University, Hangzhou, China; ^2^ Department of Orthopedic Surgery, School of Medicine, Johns Hopkins University, Baltimore, MD, United States; ^3^ Zhejiang Rehabilitation Medical Center, Zhejiang, China; ^4^ Hangzhou Yingjian Bioscience & Technology Co., Ltd, Hangzhou, China; ^5^ Department of Economic and Management, University of Jinan, Shangdong, China

**Keywords:** lipoaspirate, femoral head necrosis, angiogenesis, theraputic, stem cell

## Abstract

Femoral head necrosis (FHN), one of the most popular joint diseases in the musculoskeletal system, is usually attributed to local ischemia of the femoral head. Thus, regenerating the vascularization capacity and restoring the local perfusion of the femoral head becomes an efficient therapeutic approach for FHN. We investigated the function of autologous lipoaspirate cells (LPCs) in regenerating circulation in FHN animal models and human subjects in this study. We also explored the mechanisms of why LPCs show a superior effect than that of the bone marrow-derived stem cells (BMSCs) in vascularization. Thirty-four FHN patients were recruited for the randomized clinical trial. Harris Hip Score (HHS) and digital subtraction arteriography (DSA) and interventional technique were used to compare the efficacy of LPCs treatment and vehicle therapy in improving femoral head circulation and hip joint function. Cellular mechanism that underlies the beneficial effect of LPCs in restoring blood supply and rescuing bone architecture was further explored using canine and mouse FHN animal models. We found that LPCs perfusion through the medial circumflex artery will promote the femoral head vascularization and bone structure significantly in both FHN patients and animal models. The HHS in LPCs treated patients was significantly improved relative to vehicle group. The levels of angiogenesis factor secreted by LPCs such as VEGF, FGF2, VEC, TGF-β, were significantly higher than that of BMSCs. As the result, LPCs showed a better effect in promoting the tube structure formation of human vascular endothelial cells (HUVEC) than that of BMSCs. Moreover, LPCs contains a unique CD44^+^CD34^+^CD31^−^ population. The CD44^+^CD34^+^CD31^−^ LPCs showed significantly higher angiogenesis potential as compared to that of BMSCs. Taken together, our results show that LPCs possess a superior vascularization capacity in both autonomous and paracrine manner, indicating that autologous LPCs perfusion *via* the medial circumflex artery is an effective therapy for FHN.

## Introduction

Femoral head necrosis (FHN) is one of the destructive bone diseases in orthopedic, which will finally result in pain, destruction, and dysfunction of the joints (Narayanan et al., 2017). As reported, the risk factors could be various including alcohol abuse, high exposure to steroids, overweight, vasculopathy, and family genetic factors, meanwhile, the vascular interruption, intravascular occlusion, and extravascular compression also could be the mechanical risks. It is more common in men aged 25 to 44 and women aged 55 to 75 prevalently (Hernigou et al., 2006; Colomb-Lippa, 2014; Villa et al., 2016). The traditional treatments for FHN include conservative therapies, such as weight restriction, bisphosphonate administration, and surgeries, such as core decompression, bone grafting, total hip arthroplasty (THA) (Arbeloa-Gutierrez et al., 2016; Ye et al., 2017; Sultan et al., 2018). However, there is still no satisfactory therapeutic strategy to prevent the femoral head from collapse, arthroplasty, and promote the function of the hip joint because the insufficient femoral head terminal blood circulation and constant biomechanical challenge from the hip joint (Mont et al., 2015).

The primary blood supply of femoral head comes from the medial circumflex artery (Lavigne et al., 2005; Kamal et al., 2012). S. Sevitt and R. G. demonstrated that nearly sixty percent of blood is supplied by the medial circumflex artery in which the most important arterial branch was identified as the superior retinacular arteries (Sevitt and Thompson, 1965). Based on current knowledge of femoral head necrosis, ischemia is an early pathophysiological manifestation of FHN which directly initiates bone necrosis as reported by Enrique Guerado and his colleague. During the ischemia phase, vascular supply is interrupted related to endothelial dysfunction followed by a critical sign of bone cell apoptosis/necrosis. In this case, new vessels are needed to pierce again to support the regeneration phase (Lazaro et al., 2015; Guerado and Caso, 2016). Insufficient blood supply may lead to failure of regeneration and contributes to femoral head collapse eventually. Mesenchymal stem cell (MSC) is a regenerable lineage which has the potential to proliferate *in vitro* as well as the multipotent differentiation capabilities. Previous report showed that MSCs have the potential to differentiate to vascular cells (Lau et al., 2014), making them an attractive source for cell-based regenerative medical therapies in an attempt to restore the blood supply in FHN conditions (Jones et al., 2008; Lau et al., 2014; Papakostidis et al., 2016).

The sources of MSCs were reported various, such as bone marrow, peripheral blood, fat tissue (Lim et al., 2013). Although bone marrow MSCs have been frequently used in clinics, bone marrow procurement is painful for donors, and anesthesia is required (Gangji et al., 2011; Jin et al., 2012). On the contrary, adipose tissue has easier accessibility, higher productivity, and similar differentiation potential relative to bone marrow MSCs (Zuk et al., 2001). It was reported that the adipose MSCs show a greater angiogenesis potential (Lu et al., 2018). Human lipoaspirate cells (LPCs) isolated from human adipose tissue by suction-assisted lipectomy, contain mesodermal stem cell populations which perform multiple differentiation potential (Mizuno and Hyakusoku, 2003). Thus, LPCs become an important alternative source for mesenchymal tissue regeneration including avascular femoral head necrosis treatment (Pak et al., 2014). It has been shown that injecting or grafting LPCs significantly promote angiogenesis (Arderiu et al., 2019; Yu et al., 2020; Xia et al., 2021), but the mechanism is still not clear.

MSC implantation methods are various. Core decompression (CD) combined with MSC transplantation has become a common approach for FHN treatment (Tabatabaee et al., 2015), in which the MSCs were directly delivered into the necrotic region of the femoral head following CD (Ma et al., 2014). As it was reported that the Western Ontario and McMaster Universities Arthritis Index (WOMAC) and visual analogue scale (VAS) were significantly improved by the combination treatment, and ameliorated necrotic areas determined by MRI (Rastogi et al., 2013; Mao et al., 2015; Wang et al., 2019). However, the conflict risk of core decompression such as perforations in the femoral head, or femoral head fracture limits its clinical application. Cytokine pretreated or gene modified MSCs transplantation and tissue engineering technological MSCs transplantation are the other methods in FHN treatment (Xu et al., 2020). In these methods, BMP-2, VEGF-165, bFGF-2 pretreated or modified MSCs, and gelatin hydrogel, artificial bone tissues combination with MSCs as reported recently (Hang et al., 2012; Xu et al., 2020). Although a majority of these approaches showed a certain level of effect on promoting the repair of the necrotic area, the biosecurity and ethical concerns limit their further clinical application (Jones et al., 2008; Xu et al., 2020). In this case, arterial perfusion of MSCs draws attention as a practical treatment strategy for FHN which holds the promise to restore the femoral head blood supply. MSCs have been reported to promote arteriogenesis by releasing vascular endothelial growth factor (VEGF) and basic fibroblast growth factor (bFGF) (Narayanan et al., 2017; Xu et al., 2020). Our previous study demonstrated that both angiogenesis and vascular structure in the necrotic region of femoral head could be promoted by MSCs perfusion, of which the mechanism including the expression of VEGF and increasing microvessel density (Jin et al., 2012; Mao et al., 2015; Jin et al., 2016).

In this study, we investigated the therapeutic effect of combined LPCs therapy with medial circumflex artery perfusing approach in human subjects with FHN And FHN animal models. We identified a sub-cluster of LPCs that possesses a high angiogenesis potential, which highlights the advantages of LPCs therapy over BMSCs treatment.

## Materials and methods

### Patients and clinical methods

This study was performed at the Zhejiang Provincial Hospital of Traditional Chinese Medicine from October 2014 to January 2017 and registered as a randomized controlled trial in the Chinese Clinical Trial Registry. The ethics document was provided by The Ethics Committee of this hospital. Our inclusion criteria including the diagnostic of FHN, stage I and II according to the Association Research Circulation Osseous (ARCO), and without other physical disease. The exclusion criteria including the loss of following, the deficiency of images and the patient who accept the total hip joint replacement surgery. And all the patients received an informed consent file.

A total of thirty-four patients met the final inclusion criteria who were randomly grouped by the envelope method as control group or LPCs treatment group respectively (Table 1). 1) control group: patients received with contrast medium and saline as vehicle treatment (*n* = 8). 2) treatment group: patients received with contrast medium and lipoaspirate cells subsequently (*n* = 26). There is no significant difference in gender distribution. It included three females and five males in the control group, relative to seven females and nineteen males in the treatment group (*p* = 0.666). Meanwhile, the average age was 48.1 years (range, 29–59 years) in the control group, relative to 46.2 years (range, 35–53 years) in the treatment group, which show no significant neither (*p* = 0.0.442). And there is no significant difference in the distribution of the ARCO stage in groups (*p* = 0.565).

**TABLE 1:** Information of included patients.

Characteristics	Treatment	Controls	*p* Value
Number of patients	26	8	N/A
Age (years)	46.2 (±2.8)	48.1 (±6.7)	0.442
Male: Female	19:7	5:3	0.666
ARCO stage			
I	8	2	0.565
II	18	6	

Patients involved in this study received digital subtraction arteriography (DSA) for vessel detection and lipoaspirate cells or vehicle infusion into the medial circumflex artery nearly once a month, under the procedure as we previously reported (Mao et al., 2015). Briefly, Seldinger femoral artery puncture was used under local anesthesia. The vessel images of the femoral head were obtained by injecting a contrast medium. Then, we injected LPCs or vehicle *via* the same route. Further, their Harris Hip Score (HHS) assessment occurred within 12 h before each DSA examination, followed by the previous study (Mao et al., 2015). The higher the score, the better the outcome for the individual. Finally, the fused vessel area analysis in the femoral head will be determined by ImageJ software.

The LPCs were obtained from the patient’s abdomen subcutaneous fat following the protocol as Dr. Zuk showed previously by Hangzhou Yingjian Biological Technology Co., Ltd (Zuk et al., 2001). In brief, we infuse saline and the vasoconstrictor epinephrine into the subcutaneous through a hollow blunt-tipped cannula. Then, wash and digest the lipoaspirate tissue at 37°C for 30 min with 0.075% collagenase. Next, a high concentrate Stromal Vascular Fraction (SVF) pellet was obtained after centrifugation. The pellet was resuspended in 160 mM NH_4_Cl and incubated at room temperature for 10 min to lyse red blood cells. To distinguish LPCs from the SVF, we collected and filter the SVF, further incubated it overnight, and wash it extensively with PBS to remove residual red blood cells. The resulting cell population was processed as LPCs, which the phenotypes of CD13, CD29, CD44, CD73, CD90, CD34, CD31, CD45 were determined by flow cytometry, will be cultured *in vitro* to expand the cell counts. Finally, 25 ml cell suspension, which contains more than 1 × 10^7^ cells, determined by cell counting after culturing, was infused for each patient at the speed of 1–5 ml per minute.

### Real-time PCR analysis

Total RNA was isolated from cultured cells using the RNA isolation RNeasy mini kit (Qiagen, United States), and followed the instructions from manufacturer. The reverse transcription was determined by the PrimeScript RT Master Mix reagent kit and followed the protocol from the manufacturer (Takara, United States). And the gene of VEGF, FGF2, VEC, TGF-β were detected by qPCR using the SYBR^®^ FAST Green Master Mix kit. The quantification was analyzed with the comparative C(t) method using GAPDH as the house keeping gene. The primers were listed below. VEGF-F: AGG​GCA​GAA​TCA​TCA​CGA​AGT, VEGF-R: AGG​GTC​TCG​ATT​GGA​TGG​CA; FGF2-F: AGA​AGA​GCG​ACC​CTC​ACA​TCA, FGF2-R: CGG​TTA​GCA​CAC​ACT​CCT​TTG; VEC-F: AAG​CGT​GAG​TCG​CAA​GAA​TG, VEC-R: TCT​CCA​GGT​TTT​CGC​CAG​TG; TGF-β-F: CCA​CCT​GCA​AGA​CCA​TCG​AC, TGF-β-R: CTG​GCG​AGC​CTT​AGT​TTG​GAC; GAPDH-F: CAT​CAC​TGC​CAC​CCA​GAA​GAC​TG, GAPDH-R: ATG​CCA​GTG​AGC​TTC​CCG​TTC​AG.

### Matrigel-based tube formation assays

The angiogenesis was determined by the tube formation assays following the manufacturer’s protocol (EMD Millipore, United States). We added 100 μl ECM gel matrix solution into each well of 24-well plate and incubated it for 30 min at 37°C and 5% CO_2_. The target cells were then seeded on ECM at a concentration of 40,000 cells and incubated in a specific medium for 24 h at 37°C and 5% CO_2_. The tube structure networks were observed using an inverted microscope. And the total length of tubes was analyzed by ImageJ software. The mouse primary LPCs used for the tube formation assays were isolated from mouse abdominal adipose tissue following the protocol above. The mouse primary BMSCs used here were purchased from the company.

### Flow cytometry

The specific cell surface antigens of LPCs were characterized by flow cytometry. The cells were incubated with fluorescence-conjugated antibodies at the recommended concentration for 30 min in ice. All the antibodies were obtained from BD biosciences, including anti-human CD29, CD44, CD73, CD90, CD34, CD31, CD45, anti-mouse CD44, CD31, CD34. Data analysis was performed with FlowJo software (FlowJo, Ashland, OR).

### Animal model and experimental design

Six mature beagle dogs weighing 14.39 ± 2.36 kg were involved in the study and were divided into two groups (Vehicle group and lipoaspirate cells group, *n* = 3 per group). According to the Guidelines for the Care and Use of Laboratory Animals, all the animals were kept and maintained by the Animal Experiment Center of Zhejiang University of Traditional Chinese medicine. FHN model established by hip dislocation and liquid nitrogen followed the method of our previous report (Jin et al., 2012). In brief, surgical dislocation was performed to expose the femoral head, and 100 ml of liquid nitrogen was applied to freeze the femoral head for a half minute. Then, we rewarmed the femoral head with 37 °C normal saline before the second frozen and closed the joint. Antibacterial prophylaxis was regulated by penicillin 40,000 UI/kg for 1 week. All the beagle dogs were kept in the cage which allows them to freely active for 2 months. The LPCs isolated from the abdomen subcutaneous adipose tissue followed the protocol mentioned above. And the LPCs and vehicle medium were perfused *via* the medial circumflex artery strictly following the protocol described above.

### Histology and immunohistochemistry analysis

Dogs were euthanized by the animal facility and the femoral heads were harvested and fixed in 10% buffered formalin for 72 h at 4°C. Then, the samples were decalcified by 0.5M EDTA (PH 7.4) for 2 months at 4°C. Samples were then embedded in paraffin for histology and immunohistochemistry experiments. Four-µm-thick sagittal-oriented sections of the paraffin-embedded joint sample were processed for hematoxylin and eosin (HE) staining and immunohistochemistry staining using a standard protocol. Firstly, the sections were prepared for primary antibodies incubation, which included primary antibodies to mouse Osteocalcin (diluted 1:100; MA1-20786, Thermo Fisher, MA, United States), overnight at 4°C and incubated with secondary HRP-labeled goat anti-rabbit antibody (diluted 1:100; ab6721, Abcam, Cambridge, United Kingdom) for 60 min at room temperature. The chromogenic reaction was revealed by DAB staining.

### Three-dimensional microcomputed tomography analysis

Micro-computed tomography analysis was performed after fixation (Skyscan 1176; Bruker μCT, Kontich, Belgium). The scanner was set at a voltage of 45 kV, a current of 500 μA, and a resolution of 18 μm per pixel. We used NRecon v1.7 for image reconstruction, CTAn 1.16.9 for data analysis, CTvol 2.3.2 for three-dimensional model visualization to analyze the parameters of the subchondral bone of femoral head. The subchondral bone was segmented and analyzed to determine the bone volume fraction (BV/TV), trabecular thickness (Tb. Th), Trabecular pattern factor (Tb. Pf).

### Angiography

We used microcomputed tomography for imaging the medial circumflex artery following MICROFIL injection. Briefly, the medial circumflex artery was exposed after euthanization, we flushed the blood with 0.9% normal saline containing 100 U ml-1 heparin sodium *via* a guidance tube in the artery. Then, we prepared and infused the MICROFIL Injection Compounds (Flow Tech, Inc, MA, United States) into the medial circumflex artery as the manufactory introduced. The femoral head samples were fixed at 4°C for 48 h, followed by decalcified and imaged by μCT.

### Statistical analysis

All the data were performed as mean ± standard deviation. t-test was used to compare the means among the two animal groups and the HHS of two patient groups. The Pearson test was used to determine the significant difference in gender and age distribution of clinical patients. The statistical tests were performed by software SPSS 22.0. *p* < 0.05 was considered to be statistically significant.

## Results

### Multiple infusions of lipoaspirate cells effectively promote femoral vascularization and hip function in FHN patients

**FIGURE 1:**
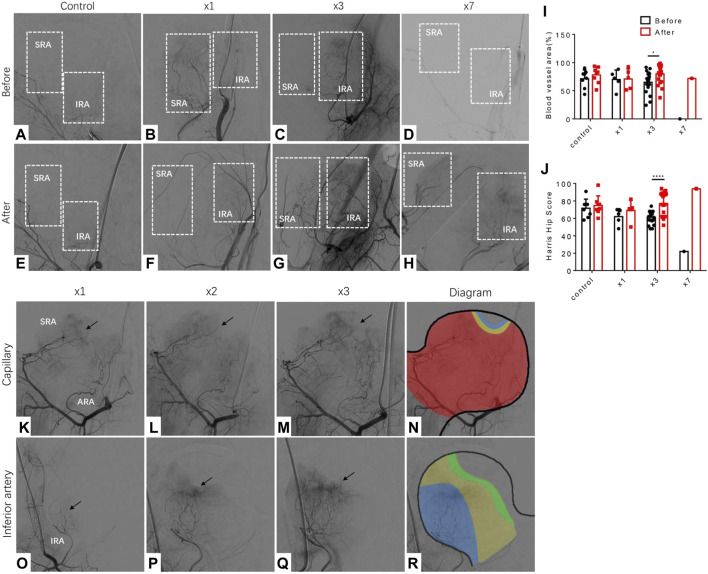
Vascularization and promotion of hip function in FHN patients received multiple LPCs infusion treatments. **(A–D)** DSA images perform the branches of the medial circumflex artery before treatment in different groups (zero-time, one time, three times, seven times, respectively). The white rectangle selects the area where distribute the branches of the medial circumflex artery or should be. SRA: superior retinacular artery, IRA: inferior retinacular artery. **(E–H)** DSA images perform the branches of the medial circumflex artery after treatment. The white rectangle selects the area where the new branches of the medial circumflex artery growth or will be. **(I)** The quantitative analysis of the blood vessel area measured in femoral head in different groups before and after treatment. **(J)** The quantitative analysis of Harris Hip score measured in four different groups before and after treatment. **(K–N)** Representative DSA images that represent the capillary network priority regeneration type along with three times treatment. Red area: original blood area; Yellow area: regenerated area after the first treatment; Blue area: regenerated area after the second treatment. **(O–R)** Representative DSA images that represent the inferior priority regeneration type along with three times treatment. Blue area: original blood area; Yellow area: regenerated area after the first treatment; Green area: regenerated area after the second treatment.

To investigate the effect of LPCs treatment, we monitored the changes of femoral head blood supply through continuous DSA images from each patient. We treated patients with LPCs *via* medial femoral circumflex artery for once, three-time or seven-time respectively based on the feedback and discussion with participants during the follow up visits after initial treatment. DSA images from patients who received one injection (*n* = 5), three injections (*n* = 20), seven injections (*n* = 1), and vehicle-treated control group (*n* = 8) were included for further analysis. The patient who received seven times treatment was also subjected to core decompression and tantalum rod transplantation (Figures 1D–H). The percentage of blood vessel area was significantly increased in the femoral head after three injections as compared to their original perfusion images before treatment. The difference of blood vessel area between before and after in one-time treatment groups did not statistically sound (Figures 1A–G). It indicated that three times treatment with LPCs infusion can significantly promote vascularization in FHN patients. Consistently, compared with the primary HHS record, we found that three-time LPCs injection significantly rescued the pain symptoms of FHN and promoted hip function relative to their baseline HHS score before treatment, while the HHS didn’t show any significant difference between the control groups or the on-time treated groups (Figure 1J). The percentage of blood vessel area and HHS score also remarkably increased after 7-time LPCs injection (Figure 1I,J). Due to the limited sample size, statistical analysis was not performed in this group. However, the X ray image analysis didn’t show significantly reverse change of bone structure in neither group but remain a relatively complete femoral head in treated groups (Supplementary Figure S1). These results indicated that multiple infusions of LPCs *via* the medial circumflex artery can significantly promote vascularization and release hip dysfunction for FHN patients.

By further analyzing the characteristics of angiogenesis induced by LPCs injection in FHN patients, we found two typical phenotypes from the twenty cases with the improved blood supply in the femoral head. Fifteen out of the twenty patients presented a capillary network regeneration priority (Figure 1K–M). The ischemia area of these fifteen cases was primarily located at the weight-bearing area, while the superior retinacular artery and inferior retinacular artery were largely intact. The diagram demonstrated the characteristics of vascularization around the weight-bearing circle (Figure 1N). The rest five cases demonstrated inferior retinacular artery regeneration priority. In these cases, we observed several small blood vessels arising from the inferior retinacular artery with bunches of the capillaries forming networks during multiple LPCs infusion (Figure 1O–R).

### The paracrine property of lipoaspirate cells promotes angiogenesis of endothelial cells

**FIGURE 2:**
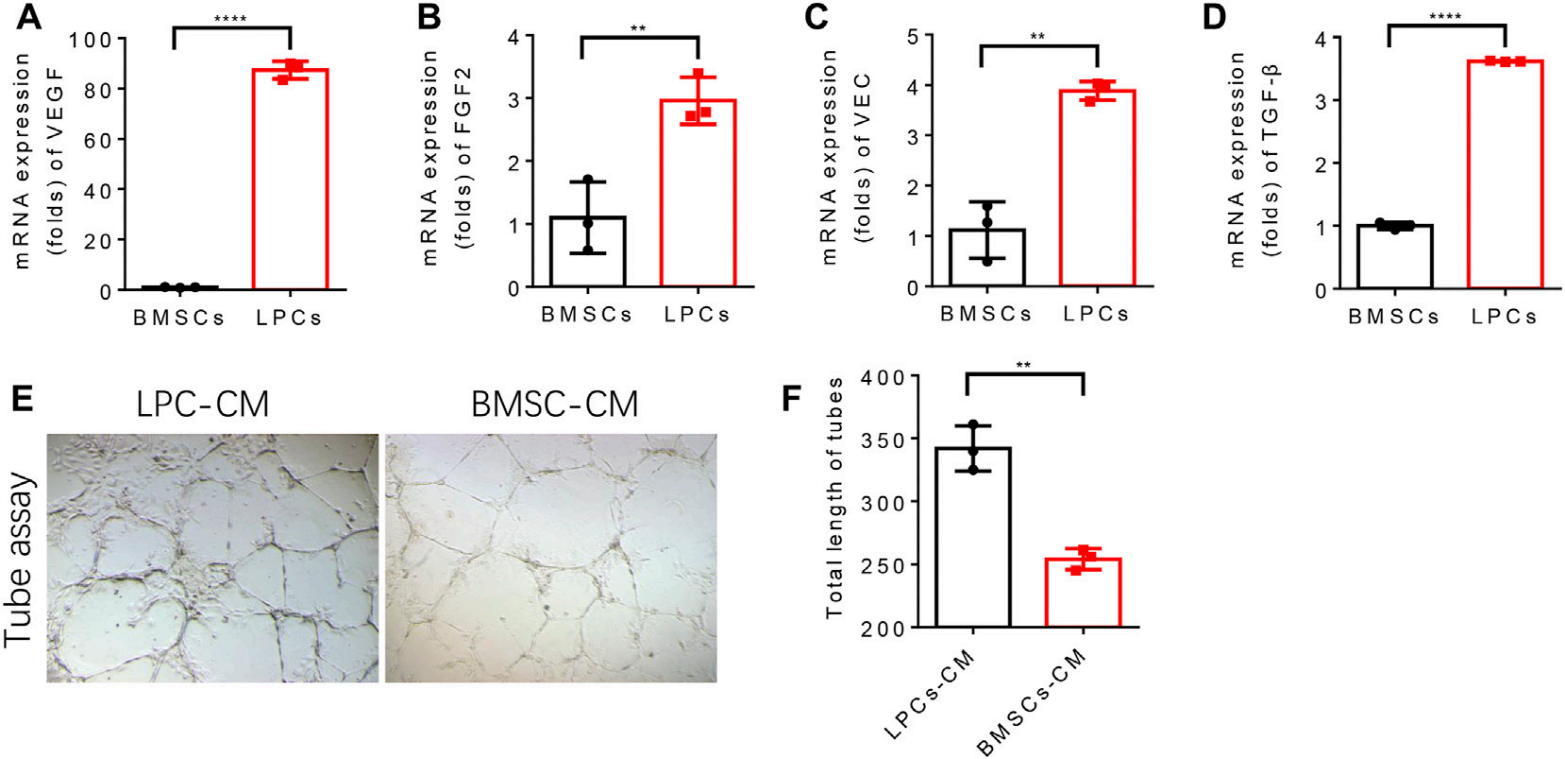
LPCs promote angiogenesis of endothelial cells by paracrine. **(A–D)** Quantitative analysis of the mRNA expression of VEGF, FGF2, VEC, TGF-β in BMSCs and LPCs. **P＜0.0001. **(E)** Representative microphotographs of capillary-like tubes formed by induced HUVEC cells on Matrigel in the presence of BMSCs or LPCs conditioned media. **(F)** Quantitative analysis of the total length of the tubes structure in BMSCs or LPCs conditioned media treated groups (Pixels). **p = 0.0015.

To examine whether LPCs possess a better angiogenesis capacity relative to BMSCs and explore the potential mechanism, we compared the autonomous angiogenesis potential and paracrine angiogenic profile between LPCs BMSCs. The mRNA levels of angiogenesis-related growth factors, including VEGF, FGF2, VEC, and TGF-β by the q-PCR experiment were compared between primary cultured mouse LPCs and BMSCs. We found that the expression levels of VEGF, FGF2, VEC, TGF-β in LPCs were significantly higher than that of BMSCs (Figure 2A–D). To further verify if LPCs can stimulate vessel formation by secreting angiogenic growth factors, we treated the human vascular endothelial cells (HUVEC) with the conditional medium collected from primary LPCs or BMSCs cultures for 2 days. Then, we seeded the pre-treated HUVEC cells in the precoated Matrigel wells and cultured them in LPCS condition medium or BMSCs condition medium, respectively. The total length of the tubes was analyzed after 24 hours. We observed a more extensive network and longer length of tube structures in the LPCs condition medium treated group as compared with that of BMSCs condition medium treated group (Figure 2E,F). This finding indicates that LPCs can substantially promote the angiogenesis of endothelial cells in a paracrine manner.

### The autonomous angiogenic properties of lipoaspirate cells relative to bone marrow-derived stem cells

**FIGURE 3:**
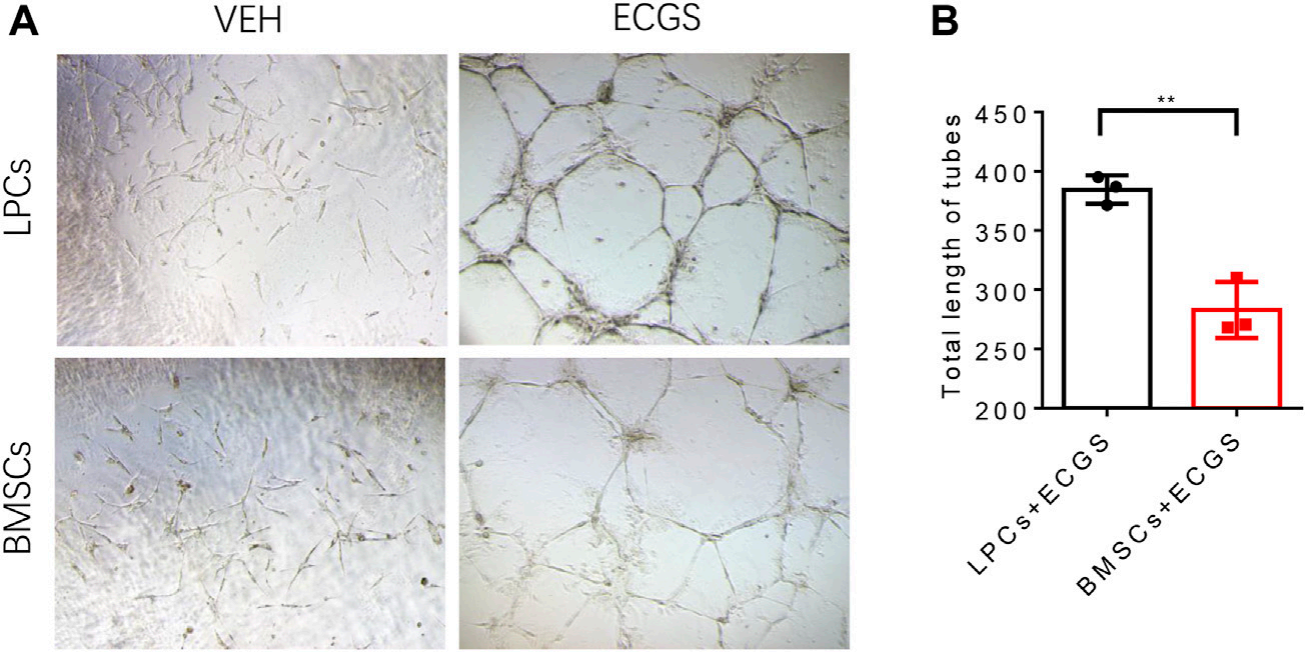
The characteristic angiogenic properties of LPCs and BMSCs. **(A)** Representative microphotographs of capillary-like tubes formed by induced LPCs and BMSCs on Matrigel in the presence of ECGS or vehicle media. **(B)** Quantitative analysis of the total length of tubes structure in the ECGS or vehicle treated groups (Pixels). **p = 0.0027.

To test the commitment of LPCs to vessel forming cells and their angiogenic potentials we compared the tube formation capacity between LPCs and BMSCs that cultured in the angiogenic medium. Both LPCs and BMSCs were cultured with endothelial cell growth supplements (ECGS) or vehicle for 10 days, and the induced cells were seeded on the Matrigel wells for the tube formation assay. We observed a significantly longer length of tube structures in ECGS stimulated LPCs relative to ECGS stimulated BMSCs (Figure 3A,B). This data suggests that the LPCs possess the capability to differentiate to an endothelial type of cells and their angiogenic potentials are higher than BMSCs.

### The CD44^+^CD34^+^CD31^−^ sub-cluster of lipoaspirate cells is the primary contributor to angiogenesis

**FIGURE 4:**
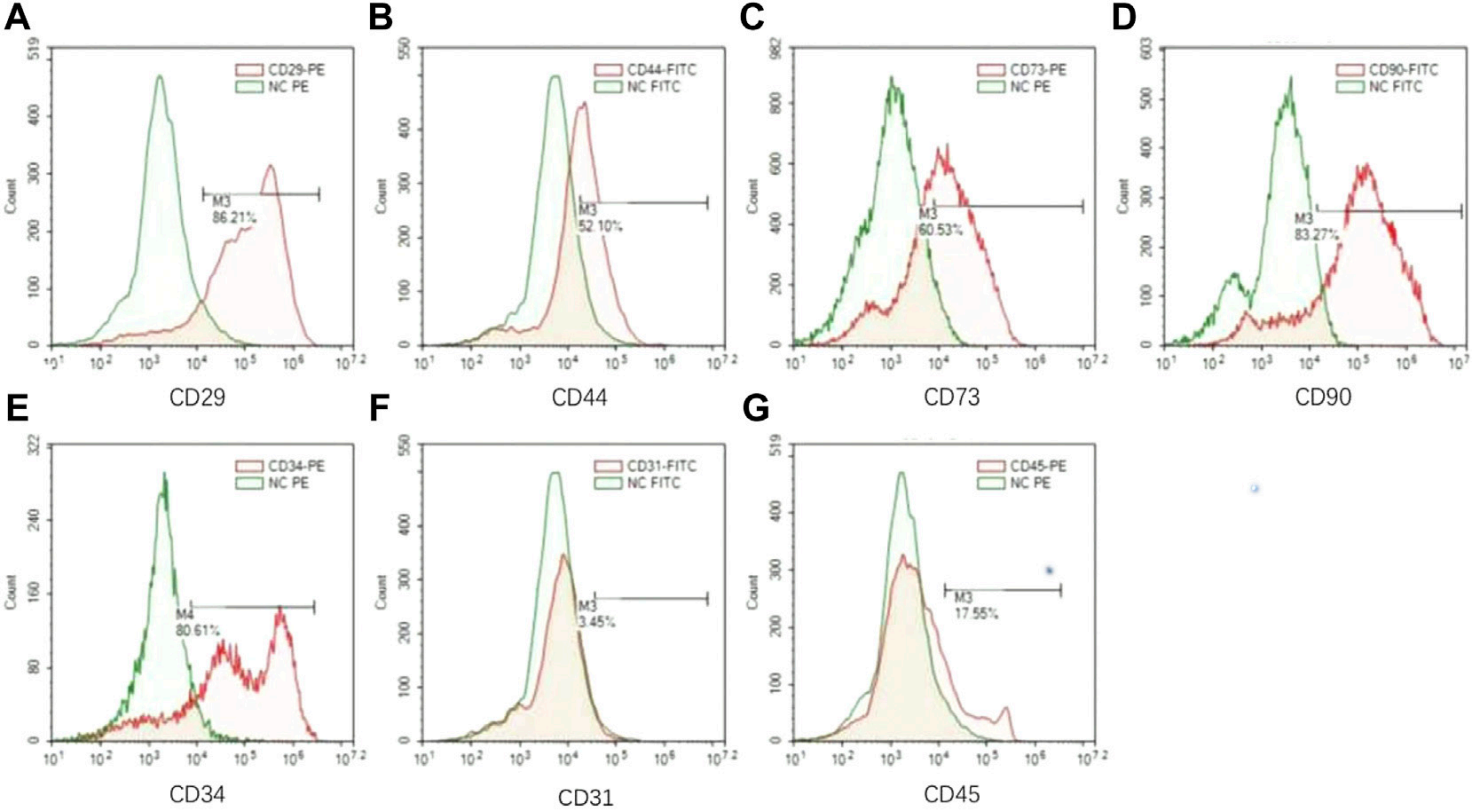
The surface markers of LPCs. **(A–G)** Flow cytometry analysis for CD29, CD44, CD73, CD90, CD34, CD31, CD45 in LPCs. Green color histogram represents isotype negtive control, red color histogram represents cells stained with fluorescent antibodies. And the positive range selected performs the representative percentage of the positive population for each marker.

We then investigated if there is a subpopulation in the heterogenic LPCs that predominantly contribute to vascular formation. We firstly analyzed the profile of surface markers in the LPCs collected from human subjects by flow cytometry. We adopted the well-accepted surface marker panel for adipose-derived mesenchymal stromal cells (e.g. CD29^+^, CD44^+^, CD73^+^, CD90^+^ CD31^−^ CD45^−^) (Rautiainen et al., 2021) to analyze these cells. The results demonstrated that there was more than fifty percent of the LPCs are positive to CD29, CD44, CD73, CD90, or CD34 (Figure4A–E), while the expression of CD31 was less than five percent and the expression of CD45 was less than twenty percent (Figure 4F,G).

**FIGURE 5:**
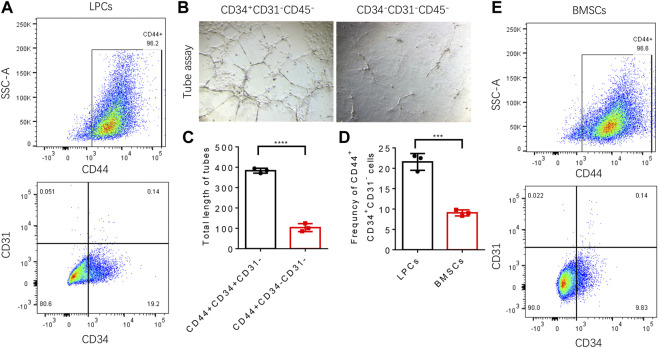
Angiogenic function of CD44^+^CD34^+^CD31^−^ LPCs. **(A)** Representative flow cytometry plots show the percentage of CD44^+^CD34^+^CD31^−^ LPCs. **(B)** Representative microphotographs of capillary-like tubes formed by induced CD44^+^CD34^+^CD31^−^ LPCs and CD44^+^CD34^−^CD31^−^ LPCs on Matrigel in the presence of ECGS media. **(C)** Total length of tubes structure (Pixels). ****P＜0.0001. **(D)** Statistic analysis of the frequency of CD44^+^CD34^+^CD31^−^ population in LPCs and BMSCs. ***P＜0.0001. **(E)** Representative flow cytometry plots show the percentage of CD44^+^CD34^+^CD31^−^ BMSCs.

Strikingly, more than eighty percent of LPCs we harvested from human subjects were CD34^+^ (Figure 4F,G). It was reported that CD34 is highly expressed in adipose-derived stem cells and this population gradually diminished during serial passage (Mitchell et al., 2006). It has been shown that the CD34^+^ adipose-derived stem cells have a superior potential for neovascularization (Planat-Benard et al., 2004; Torsney et al., 2007; Campagnolo et al., 2010). To validate whether CD34 positive population in LPCs plays the primary role in angiogenesis, we sorted the CD44^+^CD34^+^CD31^−^ and CD44^+^CD34^−^CD31^−^ LPCs harvested from mouse adipose tissue using FACS assay (Figure 5A). The angiogenic property of CD44^+^CD34^+^CD31^−^ and CD44^+^CD34^−^CD31^−^ LPCs were evaluated by culturing these two subclusters in ECGS medium for tube formation assay. The total lengths of tube structures were calculated after seeding on precoated Matrigel wells for 24 hours. We found that CD44^+^CD34^+^CD31^−^ LPCs formed a better tubing network than that of CD44^+^CD34^+^CD31^−^ LPCs. The total length of tubes in the CD44^+^CD34^+^CD31^−^ group was significantly longer than that of the CD44^+^CD34^−^CD31^−^ population (Figure 5B,C). Interestingly, the frequency of CD44^+^CD34^+^ CD31^−^ population in BMSCs was significantly lower than it in LPCs (Figure 5D,E). This finding further highlights the importance of the CD34^+^ population in LPCs angiogenesis and may explain the better effect of LPCs in angiogenesis than that of BMSCs.

### Lipoaspirate cells infusion effectively improve bone structure in canine FHN model

**FIGURE 6:**
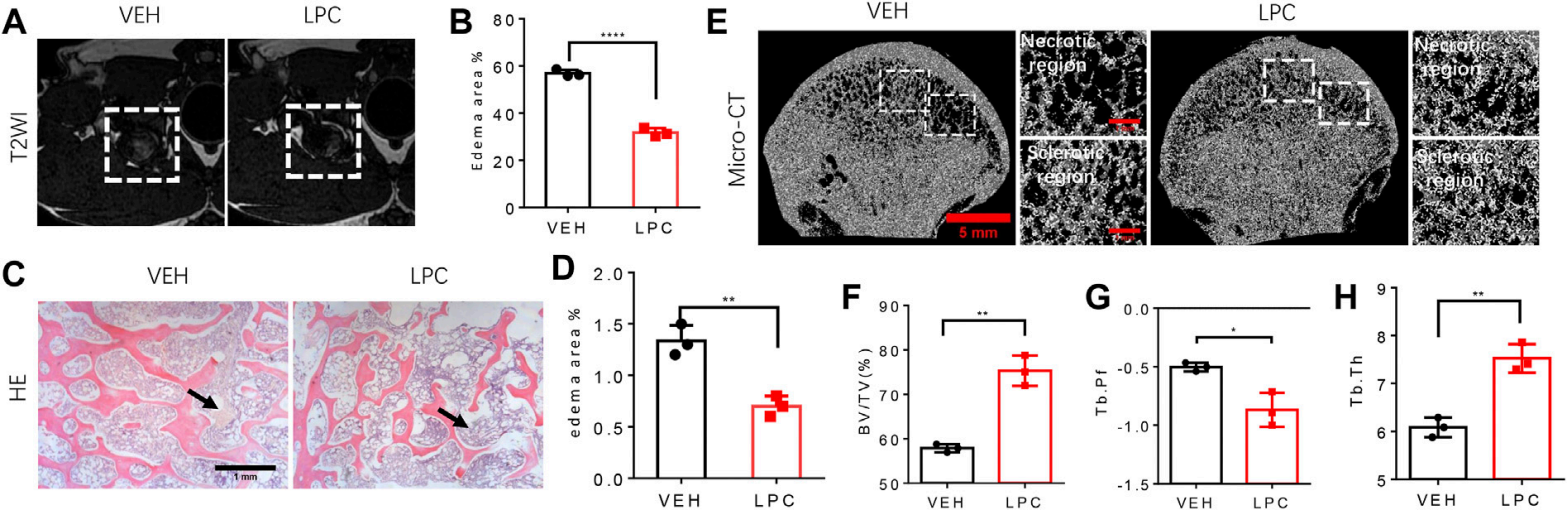
The function of LPCs in bone structure improvement. **(A)** T2WI images of the femoral head from control group and LPCs-treated group. **(B)** Quantitative analysis of edema area of two groups. ****P＜0.0001. **(C)** HE-staining show the edema degree in the medial position of femoral head in control group and LPCs-treated group, and the black arrow point at the edema area. **(D)** Quantitative analysis of edema area in control group and LPCs-treated group. **p = 0.0039. **(E)** Representative 3-dimensional micro-computed tomography (μCT) image of coronal view of the femoral head in control group and LPCs-treated group. The white frame in the subchondral bone region, point out the necrotic region, and sclerotic region, respectively. **(F)** Quantitative analysis of bone volume /tissue volume (BV/TV) ratio, **p = 0.001, **(G)** trabecular pattern factor (Tb. Pf), *p = 0.0133, **(H)** trabecular thickness in control group and LPCs-treated group. **p = 0.0023, n = 3.

To test whether LPCs treatment can rescue the bone pathologies of FHN, we examined the bone microstructure of the femoral head with or without LPCs treatment in a canine FHN model. MRI scan was performed 8 weeks after surgery to verify the success of the model establishment. We observed high-intensity signals in the T2W1 images of vehicle-treated animals. These high-intensity signals were barely observed in the femoral head after lipoaspirate cells treatment, indicating reduced edema in the LPCs treated group relative to the vehicle group (Figure 6A,B). Indeed, the edema area in the bone marrow of the femoral head was significantly decreased in the LPCs treated animals than the vehicle-treated group as illustrated by HE-staining. (Figure 6C,D). We then examined femoral head bone structure using three-dimensional microcomputed tomography (μCT) analysis in the FHN canine models. We found that the overall structure of the femoral head was significantly improved by LPCs treatment as compared to that of the vehicle-treated animals. The bone volume significantly increased in the LPCs treated group compared with vehicle controls. Higher bone volume/tissue volume ratio (BV/TV) and trabecular thickness (Tb. Th) in the LPCs treated group suggests a higher bone formation rate than that of the vehicle treated FHN dogs. Lower trabecular pattern factor (Tb. Pf) in the LPCs group indicates a better trabeculae connection and better microarchitecture in the LPCs treated group relative to the control group (Figure 6E–H). This finding indicates LPCs treatment improved the fine structure of the subchondral bone in the FHN canine model.

### Infusion of lipoaspirate cells promotes vascularization and osteocyte generation

**FIGURE 7:**
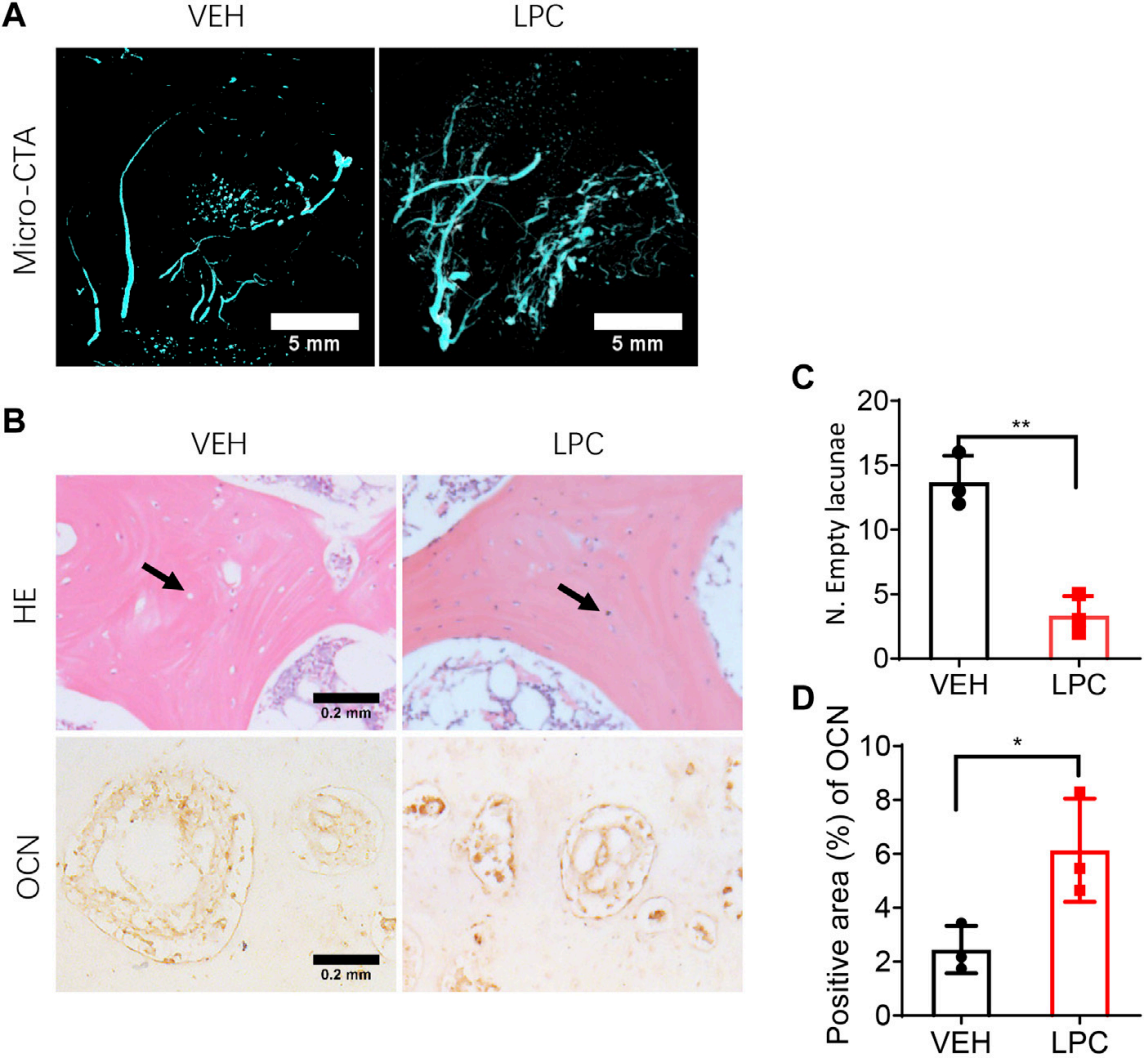
The effect of LPCs in vascularization and osteocyte generation. **(A)** Representative three-dimensional microcomputed tomography angiography (μCTA) images in the femoral head in control group and LPCs-treated group. **(B)** HE staining and immunohistochemical staining for Osteocalcin for both vehicle and LPCs cells treated groups. The black arrow shows osteocyte and lacunae. **(C–D)** Quantitative analysis of empty lacunae number and positive area of OCN. **p = 0.0023, *p = 0.0389, *n* = 3.

To test whether restoring the blood supply can reverse bone necrotic pathology in FHN, we parallelly examined the bone formation status and vascularization in the femoral head with or without LPCs treatment in the canine FHN model. We performed three-dimensional microcomputed tomography angiography (μCTA) in vehicle treated and LPCs treated animals. Both the superior retinacular artery and inferior retinacular artery showed aberrant interruption and broken in vehicle treated FHN dogs. LPCs treatment substantially rescued avascular interruption relative to the vehicle group, similar to what we observed in the human subjects (Figure 7A). To investigate whether LPCs treatment rescued bone necrosis, we performed HE staining and found that the number of empty lacunae in bone tissue was significantly lower in the LPCs treatment than that of the vehicle control group (Figure 7B,C). The osteoblast cell number was significantly greater in the LPCs treated FHN dogs as compared to that of the vehicle-treated dogs as illustrated by osteocalcin staining at the necrotic field (Figure 7B–D). These data indicate that LPCs can rescue bone necrosis and improve bone formation *via* vascularization and osteogenesis.

## Discussion

Ischemia is an early manifestation of FHN. Reduced blood supply is one of the primary contributors to bone necrosis occurring in the femoral head of FHN patients (Hauzeur et al., 1991; Guerado and Caso, 2016). However, there is no ideal revascularization measure available in clinics for FHN treatment. Enrique Guerado and his colleague summarized the location and importance of the ischemia phase in FHN (de Sanctis and Rondinella, 2000; Guerado and Caso, 2016). Vascular supply is interrupted at the beginning, with a critical sign of dead bone cells. Then the stem cells around the necrotic area play an essential role in the regeneration phase. In this phase, new vessels pierce again and follow the posterolateral direction as the medial circumflex artery to support tissue repairment (Guerado and Caso, 2016). Restoring of the blood supply to the necrotic area is not only essential for stem cell replenishment but also provides critical growth factors and nutrients for bone regeneration. In our study, the purpose of LPCs treatment *via* circumflex artery is to strengthen the positive physiopathological feedback loop. We examined the re-vascularization and bone formation at the necrotic region in parallel after LPCs treatment. We also identified a unique subpopulation of LPCs that has high angiogenesis potential.

The medial circumflex artery is the primary source of femoral head blood supply. In 1965, S. Sevitt and R. G. reported that the medial circumflex artery supplies nearly two-thirds of blood in the femoral head. The superior retinacular arteries were the most critical arterial supply to the head. However, in our current study, we found that the avascular phenotype presented randomly from superior retinacular arteries to inferior retinacular arteries and even the entire circumflex femoral arteries. Interestingly, after thoroughly analyzing the consecutive DSA images we found two predominant characteristics of vascularization during the vascular regeneration phase in FHN patients, including the capillary network priority and inferior priority types. Restoring blood supply through either the capillary network priority or inferior priority types improved the HHS score. It indicates that the relationship between vessel regeneration and hip joint function could be complicated and further investigation is needed. The characteristics of angiogenesis under the LPCs therapy may provide a reference for further basic and clinical investigation.

Our previous study has demonstrated that BMSC injection through circumflex artery had a similar or even better outcome as combined concentrated autologous BMSCs injection with core decompression in improving the outcome of vascularization in FHN patients with early-stage osteonecrosis of the femoral head. In this study, we further investigated if LPCs have a better angiogenic potential than BMSCs. We found that LPCs can promote angiogenesis in both autonomous and paracrine manners. Particularly, we identified a unique population, CD44^+^CD34^+^CD31^−^ cells, that predominantly exists in LPCs but not BMSCs. This subcluster of LPCs has a superior potential for angiogenesis.

The angiogenesis property of adipose-derived stem cells was well-reported (Miranville et al., 2004; Fischer et al., 2009; Ning et al., 2009). A different protocol may render different cell compositions when collecting these high heterogeneous adipose-derived stem cells. To date, the exact sub-population of this mixture of multipotential cells remains to be elucidated. Evelyn Torsney identified a small number of progenitor cells with variable expression of CD34, Sca-1, c-kit, and VEGFR2 markers while negative to CD133 as the source of endothelial progenitor cells. Several reports identified the CD34^+^CD31^−^ cells, with or without other markers, such as VEGFR2, TIE2, exhibited as endothelial progenitor cells in adipose-derived multipotential cells (Alessandri et al., 2001; Planat-Benard et al., 2004; Torsney et al., 2007). Based on the previous study, we further narrow down the population with high angiogenic potential as CD44^+^CD34^+^CD31^−^ cells. Moreover, we found that this population gradually diminished after several passages during the culture of LPCs, which is consistent with the previous report (Yoshimura et al., 2006). This finding suggests that multiple passages or long-time culture should be avoided when using LPCs for angiogenesis purposes.

We also validate the effect of LPCs in canines FHN model. Liquid nitrogen was used to establish the necrotic model instead of other methods because the advantages of short cycle, high repeatability, no chemical residues, limited mortality rate and won’t involve other organs (Ma J et al., 2022). The limitation of this study is the sample size could be enlarged in the future and more patients in different stages will be involved. And the control group we designed for the clinical study was treated with saline, and the images were collected months after it based on the cooperation of the patients. The statistical analysis of blood vessel reconstruction and hip joint function was compared with not only this control group but also the patients themselves before treatment. And the conclusion of the effect came from the comparison before and after treatment. This study could be an important reference for further investigation and clinical treatment for FHN patients.

## Data Availability

The original contributions presented in the study are included in the article/Supplementary Material, further inquiries can be directed to the corresponding authors.
