# Supplementary figures and images for: The therapeutic effect of adipose-derived lipoaspirate cells in femoral head necrosis by improving angiogenesis
Source: Front Cell Dev Biol. 2022 Oct 18;10:1014789. doi: 10.3389/fcell.2022.1014789 (PMC9624280; doi:10.3389/fcell.2022.1014789)

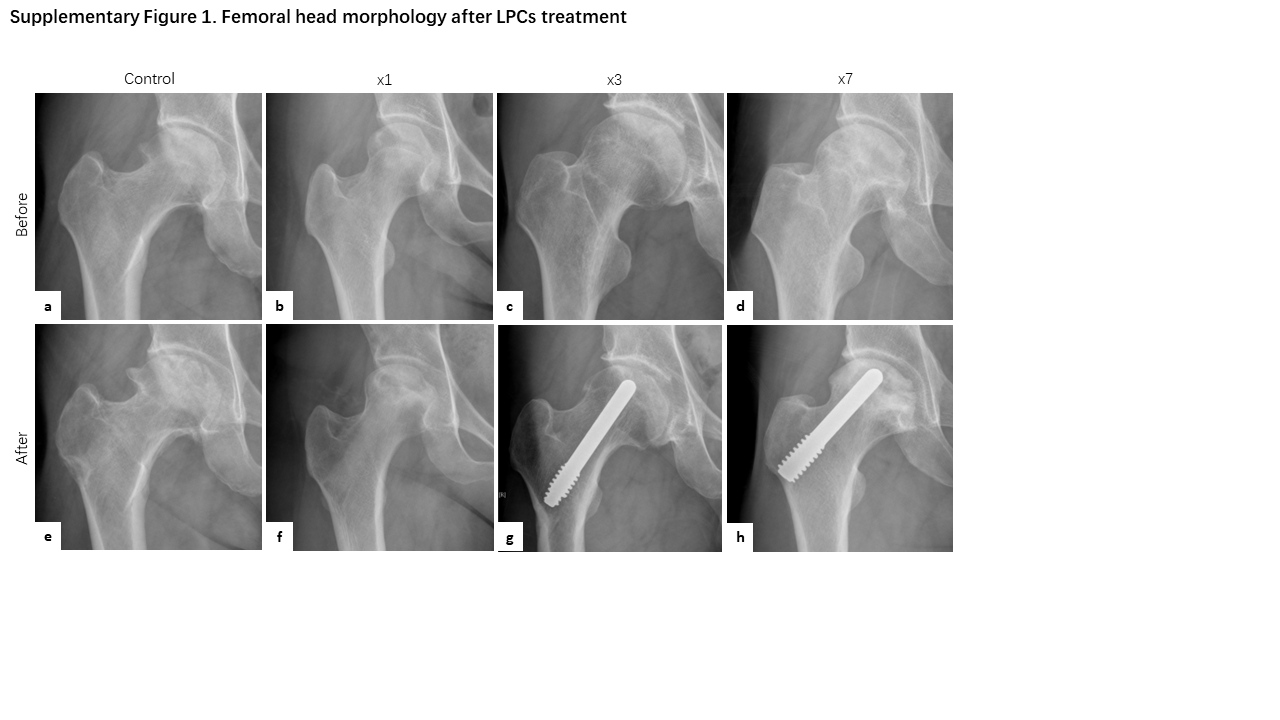

Supplement: Supplementary file 1 [file Image1.TIF]
